# Awareness of mental disorders and their risk factors - a nationwide cross-sectional survey among adults in Poland

**DOI:** 10.3389/fpsyt.2025.1599683

**Published:** 2025-05-26

**Authors:** Aleksandra Lewandowska, Andrzej Silczuk, Paulina Mularczyk-Tomczewska, Aneta Duda-Zalewska, Mateusz Jankowski, Mariusz Gujski

**Affiliations:** ^1^ Children Psychiatry Unit, Specialized Psychiatric Health Care Centre in Lodz, Lodz, Poland; ^2^ Department of Environmental Psychiatry, Faculty of Life Sciences, Medical University of Warsaw, Warsaw, Poland; ^3^ Department of Public Health, Medical University of Warsaw, Warsaw, Poland; ^4^ Department of Population Health, Centre of Postgraduate Medical Education, Warsaw, Poland

**Keywords:** mental health, mental disorders, awareness, knowledge, risk factors

## Abstract

**Introduction:**

Currently, there are no nationwide cross-sectional studies on public awareness of mental disorders in Poland. This study aimed to identify factors associated with the awareness of mental disorders and their risk factors through a nationwide cross-sectional survey of adults in Poland.

**Methods:**

This cross-sectional study was conducted between March 8 and 10, 2025, using the computer-assisted web interview (CAWI) technique. The questionnaire included questions on self-reported awareness of mental health, awareness of 9 mental disorders, and 12 mental disorder risk factors.

**Results:**

The study population comprised 1114 adults aged 18–96 years, with 54.7% being female. The majority of respondents (47.2%) reported a moderate level of awareness of mental disorders. Depression (82.6%) was the most recognized mental disorder among adults in Poland. Traumatic experiences in the past (76.8%) were the most commonly recognized risk factor for mental disorders, followed by genetic predisposition (64.5%) and difficulties in family relationships (64.5%). Factors significantly associated with a rather good/very good level of awareness of mental disorders included being under 60 years of age (p<0.05), living in cities with more than 20,000 residents (p<0.05), having higher education (aOR=1.77 [1.23-2.55]; p=0.002), and having a family history of mental disorders (aOR=2.69 [1.86-3.89]; p<0.001).

**Conclusions:**

The level of awareness of mental disorders among adults in Poland is low, and significant differences in awareness were observed based on sociodemographic variables. Understanding these disparities is crucial for tailoring effective public health campaigns and informing national mental health strategies aimed at early detection, destigmatization, and equitable access to care.

## 1 Introduction

According to the World Health Organization (WHO), health is the complete physical, mental and social well-being of a person, and not just the absence of disease or disability (1). Mental health is at the forefront in the global and European context as one of the pillars of public health, economic stability and social well-being. At the same time, it remains a fact that mental health problems constitute a significant burden for individuals, their relatives, the health care system and the entire society (2). In Poland, the results of a comprehensive epidemiological study on the mental health of society and its determinants conducted before the COVID-19 pandemic (EZOP II) showed that 26.46% of Polish men and women struggled with a mental disorder at least once in their life, of which 16.07% with neurotic disorders and 4.65% with mood disorders (3). According to the Global Burden of Disease Study (GBD), Poland loses 1,259 healthy life years per year due to mental illness (per 100,000 inhabitants), with the two most common conditions being anxiety disorders and depression, responsible for 3,082.5 and 1,192.1 cases per 100,000 inhabitants, respectively (4). The WHO European Health Equity Status Report 2019 showed that there is a significant relationship between socio-economic factors and mental health disorders as well as mental health inequalities (2). Similarly, poor mental health adversely affects productivity, the ability to achieve one’s life goals, and the economic situation.

A recent WHO report revealed that around 1 in 8 people worldwide are living with a mental disorder, with anxiety and depressive disorders being the most prevalent (5). Depression is expected to be a major contributor to the global burden of disease by 2030. Depression currently causes more disability in young people aged 10 to 24 than any other disease (5). Around 7.3% of the global burden of disease is attributed to mental and behavioral disorders. Most of this burden is attributed to unipolar depressive disorders, as well as anxiety, psychotic and substance use disorders (4, 6). Currently, around 450 million people suffer from mental disorders, and it is predicted that one in four people worldwide will be affected by a mental disorder at some point in their life (6). Mental disorders are one of the leading causes of ill health and disability worldwide (7). People with mental disorders experience disproportionately higher rates of disability and mortality (8). People with major depressive disorder and schizophrenia had a 40 to 60% higher risk of premature death than the general population (9). Between 1990 and 2019, the global number of disability-adjusted life years (DALYs) attributable to mental disorders increased from 80.8 million to 125.3 million (10). The share of global DALYs attributable to mental disorders increased from 3.1% to 4.9%. Mental disorders remain a leading cause of global health burden. Rates of burden measured in disability-adjusted life years (DALYs) were evident across all age groups. They occurred before the age of five in people with intellectual disabilities and autism spectrum disorders, and in older age in people with depression, anxiety disorders, and schizophrenia. Moreover, after considering the impact of the COVID-19 pandemic on population health (among the 25 leading causes), since 2010, age-standardized DALY rates have increased the most for anxiety disorders – by 16.7%; depressive disorders – by 16.4%; and diabetes – by 14.0% (11).

Mental health literacy, defined as “knowledge and attitudes about mental disorders that help in their recognition, treatment and prevention” is low worldwide, but particularly low in developing countries (12). Mental disorders are complex health problems that are influenced by biological, psychological and environmental factors. Understanding the risks associated with these disorders can help in their prevention and early recognition of symptoms. Researchers emphasize that lack of sufficient knowledge about mental disorders increases the risk of negative effects of developing conditions, delays effective help-seeking and treatment, and increases the risk of stigmatization (13). Currently, there are no nationwide cross-sectional studies among adults in Poland assessing awareness of mental disorders and risk factors. Raising awareness of risk factors associated with mental disorders is crucial for preventing their development and improving the quality of life of people struggling with mental problems. The National Mental Health Program for 2023–2030 is being implemented in Poland and is a strategic framework for actions aimed at improving the mental health of society (14). One of the key elements of the program is prevention, i.e. preventing the occurrence of mental disorders and promoting mental health. The implementation of the National Mental Health Program aims not only to reduce the number of new cases of mental disorders, but also to improve the quality of life of people struggling with these problems and their integration into society.

Nationwide cross-sectional surveys on public awareness of mental disorders and risk factors for mental disorders may inform healthcare professionals and policymakers on the level of knowledge on mental disorders and point out further educational needs.

Therefore, this study aimed to identify factors associated with the awareness of mental disorders and their risk factors in a nationwide cross-sectional survey among adults in Poland.

## 2 Material and methods

### 2.1 Study design and population

This study was carried out following a cross-sectional model, with data collected between 8 and 10 March 2025 using the Computer-Assisted Web Interview (CAWI) technique. To ensure a nationwide representation of adults in Poland, the study contracted a public opinion survey company [Arianda Poland’s Research Panel (15)] to collect data. The online questionnaire was hosted on a research platform managed by the company, and invitations to participate were sent via e-mail and text message to verified respondents from a pool of over 100,000 individuals. Each respondent received an invitation to participate in the survey via e-mail and text message.

While the sampling strategy was stratified by gender, age, and size of residence, ensuring demographic diversity, it is important to note that the use of CAWI may limit participation from groups without internet access, including older adults, rural populations, and those with limited digital literacy. The sampling method enabled the selection of a nationally representative group, based on demographic data from Statistics Poland (GUS), the central institution responsible for official statistical reporting (16). However, given that over 93% of households in Poland have internet access (17) and the sampling design accounted for key demographic variables such as age, the risk of systematic underrepresentation of digitally excluded individuals—particularly older adults—is minimal. The study was thus designed to be as inclusive as possible within the CAWI framework.

Participation in this survey was voluntary and anonymous. The study procedures followed the ethical and scientific standards expressed in the Helsinki Declaration. Ethical approval of the Ethics Committee of the Medical University of Warsaw was obtained (decision AKBE/38/2025 as of 24 Feb 2025).

### 2.2 Measures

The study questionnaire was prepared based on a literature review (1–5). A series of questions on public awareness of mental disorders and their risk factors were addressed. Respondents were asked to select all mental disorders and risk factors they were aware of. However, to minimize confusion, participants were provided with clear instructions to select all that applied to their knowledge, rather than only the most relevant items. Similar methodology was presented in other studies (17–21).

Self-reported level of knowledge of mental disorders was assessed based on a 5-point Likert scale. Respondents were asked about awareness of 9 different groups of mental disorders using the following question: “Have you ever heard of mental disorders listed below? (please indicate all you have heard of)”, with a response of yes/no to each mental disorder listed. A list of the 12 most common risk factors for mental disorders was prepared and the following question was addressed to the respondents: “What do you think are the risk factors for mental disorders? (please indicate all that apply)”, with a response yes/no to each risk factor listed.

Moreover, a set of questions on sociodemographic characteristics was addressed, including gender, age, place of residence, educational level, professional status (active – employed or self-employed; passive – unemployed, retired, student), relationship status, having children, living conditions, self-declared household financial status (based on self-declared assessment, as the question on monthly income resulted in the high number of missed answers). Question on the history of mental disorders in the family was also addressed: “Does any member of your immediate family (parent, sibling, child, partner) have or has had a diagnosis of a mental disorder?” (yes/no).

### 2.3 Statistical analysis

Data were analyzed with IBM SPSS version 29 (Armonk, NY, USA). The distribution of categorical variables was presented with frequencies and proportions. Cross-tables with chi-squared tests were used to compare categorical variables. A logistic regression model was used to identify factors associated with self-reported level of knowledge on mental disorders. In bivariable logistic regression, each sociodemographic variable was considered separately. In the multivariable logistic model, each variable statistically significant in the bivariable analysis was used in the multivariable model. The strength of associations was presented with odds ratio (OR) and 95% confidence intervals (95%CI). The statistical significance level was set at p<0.05.

## 3 Results

The study population included 1114 adults aged 18–96 years, 54.7% were females. Details are presented in **Table 1**.

**Table 1:** Characteristics of the study population (n=1114).

Variable	n	%
Gender		
female	609	54.7
male	505	45.3
Age [years]		
18-29	151	13.6
30-39	217	19.5
40-49	213	19.1
50-59	217	19.5
60+	316	28.4
Place of residence		
rural area	423	38.0
city <20,000 residents	143	12.8
city 20,000-99,999 residents	218	19.6
city 100,000-499,999 residents	187	16.8
city ≥ 500,000 residents	143	12.8
Educational level		
primary	30	2.7
vocational	98	8.8
secondary	478	42.9
higher	508	45.6
Professional status		
active (employed or self-employed)	685	61.5
passive (unemployed, student or pensioner)	429	38.5
Relationship status		
married or informal relationship	779	69.9
single	335	30.1
Having children		
yes	727	65.3
no	387	34.7
Living alone		
yes	175	15.7
no	939	84.3
Self-declared household financial status		
good	524	47.0
moderate	436	39.1
bad	154	13.8
History of mental disorders in the family		
yes	235	21.1
no	879	78.9

### 3.1 Public awareness of mental disorders and their risk factors among adults in Poland

Most of the respondents (47.2%) declared a moderate level of knowledge of mental disorders (**Table 2**). In this study, a “moderate” level of knowledge was defined as a self-reported score of 3 on a 5-point Likert scale, where 1 indicated “very poor” and 5 “very good” knowledge. This level reflects general familiarity with mental disorders without in-depth understanding or recognition of a wide range of conditions. Only 2.7% of respondents declared a very good level of knowledge and 13.7% declared a rather good level of knowledge of mental disorders. Out of 9 different mental disorders analyzed in this study, depression (82.6%) was the most recognized mental disorder among adults in Poland. Moreover, over half of respondents declared awareness of addictions (74.7%), anxiety disorders (62.8%), neurodevelopmental disorders (61%), suicidal behavior (57.1%), obsessive-compulsive disorder (53.7%) and bipolar disorder (52.1%). Out of 12 different mental disorder risk factors, traumatic experiences in the past (76.8%) were the most recognized risk factors for mental disorders (**Table 2**). Moreover, over half of respondents declared that genetic predisposition (64.5%) or difficulties in family relationships (64.5%) are risk factors for mental disorders. Environmental pollution was the least (12.1%) recognized risk factor for mental disorders (**Table 2**).

**Table 2:** Public awareness of mental disorders and their risk factors among adults in Poland (n=1114).

Variable	n	%
Self-reported level of knowledge of mental disorders		
very good	30	2.7
rather good	153	13.7
moderate	526	47.2
rather bad	298	26.8
very bad	107	9.6
Have you ever heard of mental disorders listed below? (please indicate all you have heard of)		
Neurodevelopmental disorders	679	61.0
Depression	920	82.6
Bipolar disorder	580	52.1
Psychotic disorders	347	31.1
Anxiety disorders	700	62.8
Obsessive-compulsive disorder	598	53.7
Conduct disorders	361	32.4
Suicidal behavior	636	57.1
Addictions	832	74.7
What do you think are the risk factors for mental disorders? (please indicate all that apply)		
Genetic predisposition (mental disorders in the family)	719	64.5
Bacterial, viral or parasitic infections during pregnancy	188	16.9
Use of stimulants during pregnancy	357	32.0
Perinatal complications	336	30.2
Low socio-economic status	348	31.2
Environmental pollution	135	12.1
Chronic diseases, e.g. diabetes, asthma, cancer	320	28.7
Digital technologies	328	29.4
Pandemic like COVID-19	369	33.1
Traumatic experiences (physical, psychological, sexual violence, war, natural disasters)	856	76.8
Difficulties in family relationships	719	64.5
Discrimination because of gender, sexual orientation, race or social class	526	47.2

### 3.2 Sociodemographic differences in public awareness of mental disorders and their risk factors among adults in Poland

There were statistically significant differences (p<0.05) in public awareness of mental disorders, mostly by gender, age, place of residence, educational level, self-reported financial household status, and the history of mental disorders in the family (**Tables 3**, **4**). Moreover, there were statistically significant differences (p<0.05) in public awareness of mental disorder risk factors: Women were more likely than men to recognize several key risk factors, including genetic predisposition (p<0.001), low socioeconomic status (p=0.006), traumatic experiences (p<0.001), difficulties in family relationships (p=0.003), and discrimination (p=0.005). Younger adults, particularly those under 40, more frequently identified digital technologies (p<0.001), the COVID-19 pandemic (p=0.002), and social discrimination (p=0.02). Respondents from larger cities showed higher recognition of genetic predisposition (p=0.002), use of stimulants during pregnancy (p=0.02), and traumatic experiences (p=0.05) compared to those from rural areas. Higher educational attainment was consistently associated with greater awareness of nearly all assessed risk factors, including genetic predisposition (p<0.001), environmental pollution (p=0.01), traumatic experiences (p<0.001), and discrimination (p=0.01). Those reporting good household financial status and individuals with a family history of mental disorders also demonstrated significantly higher awareness across multiple domains (p<0.05 for all listed factors) (**Tables 5**, **6**).

**Table 3:** Public awareness of selected mental disorders by sociodemographic factors (n=1114) – part 1.

Variable	Public Awareness of Selected Mental Disorders									
	Neurodevelopmental disorders		Depression		Bipolar disorder		Psychotic disorders		Anxiety disorders	
	%	p	%	p	%	p	%	p	%	p
Gender										
female (n=609)	68.3	**<0.001**	87.0	**<0.001**	61.2	**<0.001**	33.2	0.1	69.6	**<0.001**
male (n=505)	52.1		77.2		41.0		28.7		54.7	
Age [years]										
18-29 (n=151)	64.2	0.8	79.5	**<0.001**	55.0	0.3	40.4	**0.003**	66.9	0.3
30-39 (n=217)	60.4		75.6		56.7		36.4		60.8	
40-49 (n=213)	58.2		78.4		46.9		32.4		60.1	
50-59 (n=217)	62.7		88.0		51.2		27.2		67.7	
60+ (n=316)	60.4		88.0		51.6		25.0		60.8	
Place of residence										
rural area (n=423)	58.6	**0.01**	81.3	0.5	45.4	**<0.001**	24.1	**<0.001**	60.0	0.2
city <20,000 residents (n=143)	52.4		79.7		46.9		26.6		60.1	
city 20,000-99,999 residents (n=218)	60.1		84.9		55.5		33.0		63.8	
city 100,000-499,999 residents (n=187)	70.6		82.4		60.4		39.0		64.2	
city ≥ 500,000 residents (n=143)	65.0		86.0		60.8		43.4		70.6	
Educational level										
primary (n=30)	46.7	**<0.001**	56.7	**<0.001**	23.3	**<0.001**	23.3	**0.003**	36.7	**<0.001**
vocational (n=98)	46.9		83.7		36.7		25.5		54.1	
secondary (n=478)	55.2		80.3		47.9		26.8		61.1	
higher (n=508)	69.9		86.0		60.6		36.8		67.7	
Professional status										
active (n=685)	59.3	0.1	79.9	**0.002**	50.1	0.09	33.1	0.07	61.6	0.3
passive (n=429)	63.6		86.9		55.2		28.0		64.8	
Relationship status										
married or informal relationship (n=779)	61.1	0.9	83.2	0.4	51.1	0.3	30.7	0.6	62.3	0.5
single (n=335)	60.6		81.2		54.3		32.2		64.2	
Having children										
yes (n=727)	60.4	0.6	83.2	0.4	49.7	**0.03**	27.0	**<0.001**	61.8	0.3
no (n=387)	62.0		81.4		56.6		39.0		64.9	
Living alone										
yes (n=175)	63.4	0.5	82.3	0.9	59.4	**0.03**	33.1	0.5	62.9	0.9
no (n=939)	60.5		82.6		50.7		30.8		62.8	
Self-declared household financial status										
good (n=524)	64.3	0.03	85.3	0.06	58.4	**<0.001**	34.9	**0.04**	65.8	**0.02**
moderate (n=436)	59.9		79.6		46.8		27.5		62.6	
bad (n=154)	52.6				45.5		28.6		53.2	
History of mental disorders in the family										
yes (n=235)	70.2	**0.001**	88.5	**0.01**	63.8	**<0.001**	40.4	**<0.001**	77.4	**<0.001**
no (n=879)	58.5		81.0		48.9		28.7		58.9	

Bold values are statistically significant.

**Table 4:** Public awareness of selected mental disorders by sociodemographic factors (n=1114) – part 2.

Variable	Public Awareness of Selected Mental Disorders							
	Obsessive-compulsive disorder		Conduct disorders		Suicidal behavior		Addictions	
Gender								
female (n=609)	63.9	**<0.001**	36.9	**<0.001**	62.2	**<0.001**	77.5	**0.02**
male (n=505)	41.4		26.9		50.9		71.3	
Age [years]								
18-29 (n=151)	58.9	**0.01**	36.4	**0.004**	59.6	0.6	71.5	**0.04**
30-39 (n=217)	58.5		36.9		59.0		70.5	
40-49 (n=213)	56.3		39.0		57.3		70.4	
50-59 (n=217)	54.8		29.0		58.5		80.2	
60+ (n=316)	45.3		25.3		53.5		78.2	
Place of residence								
rural area (n=423)	45.6	**<0.001**	26.2	**0.004**	52.2	**0.03**	74.2	0.8
city <20,000 residents (n=143)	49.0		30.1		53.1		71.3	
city 20,000-99,999 residents (n=218)	56.9		38.1		59.2		76.1	
city 100,000-499,999 residents (n=187)	62.0		36.4		63.6		75.4	
city ≥ 500,000 residents (n=143)	66.4		39.2		63.6		76.2	
Educational level								
primary (n=30)	33.3	**<0.001**	16.7	**<0.001**	40.0	**<0.001**	56.7	**0.02**
vocational (n=98)	34.7		26.5		46.9		71.4	
secondary (n=478)	49.4		27.8		51.3		72.8	
higher (n=508)	62.6		38.8		65.6		78.1	
Professional status								
active (n=685)	53.7	0.9	33.9	0.2	57.2	0.9	72.1	**0.01**
passive (n=429)	53.6		30.1		56.9		78.8	
Relationship status								
married or informal relationship (n=779)	51.9	0.06	32.6	0.8	57.5	0.7	75.6	0.3
single (n=335)	57.9		31.9		56.1		72.5	
Having children								
yes (n=727)	49.8	**<0.001**	29.6	**0.01**	55.4	0.1	75.1	0.7
no (n=387)	61.0		37.7		60.2		73.9	
Living alone								
yes (n=175)	60.0	0.07	34.3	0.6	57.7	0.9	72.6	0.5
no (n=939)	52.5		32.1		57.0		75.1	
Self-declared household financial status								
good (n=524)	58.8	**0.01**	37.6	**0.002**	62.4	**<0.001**	77.9	**0.03**
moderate (n=436)	49.1		28.0		54.6		73.4	
bad (n=154)	49.4		27.3		46.1		67.5	
History of mental disorders in the family								
yes (n=235)	66.0	**<0.001**	44.7	**<0.001**	66.0	**0.002**	80.0	**0.04**
no (n=879)	50.4		29.1		54.7		73.3	

Bold values are statistically significant.

**Table 5:** Sociodemographic differences in public awareness of mental disorders risk factor (n=1114) – part 1.

Variable	Public Awareness of Mental Disorders Risk Factors											
	Genetic predisposition		Infections during pregnancy		Use of stimulants during pregnancy		Perinatal complications		Low socio-economic status		Environmental pollution	
	%	p	%	p	%	p	%	p	%	p	%	p
Gender												
female (n=609)	69.1	**<0.001**	16.7	0.9	34.6	**0.04**	32.7	0.04	33.7	0.06	11.5	0.5
male (n=505)	59.0		17.0		28.9		27.1		28.3		12.9	
Age [years]												
18-29 (n=151)	62.3	0.07	13.9	0.1	30.5	**0.01**	31.8	**0.002**	33.8	0.4	11.9	0.2
30-39 (n=217)	60.4		19.8		36.9		30.0		35.0		12.4	
40-49 (n=213)	59.6		21.1		36.6		25.8		31.5		16.4	
50-59 (n=217)	68.2		15.7		34.6		22.6		30.9		11.5	
60+ (n=316)	69.3		14.2		24.7		37.7				9.5	
Place of residence												
rural area (n=423)	58.4	**0.002**	17.0	0.8	30.3	**0.02**	28.8	0.4	33.3	0.5	11.6	0.8
city <20,000 residents (n=143)	62.2		16.8		29.4		28.0		32.2		10.5	
city 20,000-99,999 residents (n=218)	66.1		14.7		26.6		28.0		26.6		12.8	
city 100,000-499,999 residents (n=187)	72.7		17.1		38.0		34.8		29.9		11.8	
city ≥ 500,000 residents (n=143)	72.0		19.6		40.6		33.6		32.9		14.7	
Educational level												
primary (n=30)	50.0	**<0.001**	10.0	**0.02**	20.0	**<0.001**	13.3	0.1	23.3	0.5	6.7	**0.01**
vocational (n=98)	51.0		17.3		34.7		32.7		26.5		6.1	
secondary (n=478)	60.3		13.4		24.3		28.7		31.2		10.3	
higher (n=508)	72.0		20.5		39.6		32.1		32.7		15.4	
Professional status												
active (n=685)	62.6	0.09	18.8	**0.03**	35.5	**0.002**	26.1	**0.001**	32.0	0.5	13.1	0.2
passive (n=429)	67.6		13.8		26.6		36.6		30.1		10.5	
Relationship status												
married or informal relationship (n=779)	65.0	0.7	17.7	0.3	31.8	0.8	31.2	0.3	30.2	0.2	11.9	0.8
single (n=335)	63.6		14.9		32.5		27.8		33.7		12.5	
Having children												
yes (n=727)	64.8	0.8	16.9	0.9	30.5	0.1	30.0	0.9	28.7	**0.01**	11.1	0.2
no (n=387)	64.1		16.8		34.9		30.5		35.9		14.0	
Living alone												
yes (n=175)	69.1	0.2	14.3	0.3	29.7	0.5	25.1	0.1	29.1	0.5	8.6	0.1
no (n=939)	63.7		17.4		32.5		31.1		31.6		12.8	
Self-declared household financial status												
good (n=524)	69.3	**<0.001**	16.8	0.4	34.7	0.2	32.4	0.3	30.7	0.5	11.5	0.8
moderate (n=436)	63.5		18.1		30.0		28.7		30.5		12.8	
bad (n=154)	51.3		13.6		28.6		26.6		35.1		12.3	
History of mental disorders in the family												
yes (n=235)	71.5	**0.01**	16.6	**0.9**	35.7	0.2	31.5	0.6	34.5	0.2	12.8	0.7
no (n=879)	62.7		17.0		31.1		29.8		30.4		11.9	

Bold values are statistically significant.

**Table 6:** Sociodemographic differences in public awareness of mental disorders risk factor (n=1114) – part 2.

Variable	Public Awareness of Mental Disorders Risk Factors											
	Chronic diseases		Digital technologies		Pandemic like COVID-19		Traumatic experiences		Difficulties in family relationships		Discrimination	
	%	p	%	p	%	p	%	p	%	p	%	p
Gender												
female (n=609)	29.7	0.4	29.9	0.7	36.5	**0.01**	82.4	**<0.001**	68.5	**0.003**	51.1	**0.005**
male (n=505)	27.5		28.9		29.1		70.1		59.8		42.6	
Age [years]												
18-29 (n=151)	35.1	0.3	35.8	**<0.001**	44.4	**0.002**	73.5	0.1	69.5	0.4	51.0	0.2
30-39 (n=217)	28.1		37.8		38.7		75.1		67.3		48.4	
40-49 (n=213)	24.4		30.5		29.6		72.3		65.3		40.4	
50-59 (n=217)	27.6		30.0		30.9		78.8		61.3		47.5	
60+ (n=316)	29.7		19.6		27.8		81.3		62.0		49.1	
Place of residence												
rural area (n=423)	28.4	0.5	25.5	**0.005**	29.8	0.06	72.8	0.05	63.4	0.1	44.2	0.2
city <20,000 residents (n=143)	29.4		25.9		34.3		78.3		60.8		49.0	
city 20,000-99,999 residents (n=218)	24.8		27.5		34.9		75.7		62.8		44.5	
city 100,000-499,999 residents (n=187)	29.9		39.0		30.5		82.9		64.7		51.3	
city ≥ 500,000 residents (n=143)	33.6		35.0		42.7		81.1		74.1		53.1	
Educational level												
primary (n=30)	13.3	0.06	23.3	**0.004**	23.3	0.1	60.0	**<0.001**	50.0	**0.01**	20.0	**0.01**
vocational (n=98)	24.5		27.6		32.7		63.3		59.2		41.8	
secondary (n=478)	27.2		24.5		30.3		77.4		61.3		48.3	
higher (n=508)	31.9		34.8		36.4		79.9		69.5		48.8	
Professional status												
active (n=685)	28.2	0.6	32.3	**0.01**	33.3	0.9	73.7	**0.002**	64.8	0.8	46.4	0.5
passive (n=429)	29.6		24.9		32.9		81.8		64.1		48.5	
Relationship status												
married or informal relationship (n=779)	28.1	0.5	29.4	0.9	32.2	0.3	76.8	0.9	63.5	0.3	48.4	0.2
single (n=335)	30.1		29.6		35.2		77.0		66.9		44.5	
Having children												
yes (n=727)	27.2	0.1	26.3	**0.001**	29.4	**<0.001**	77.4	0.5	62.4	**0.04**	48.0	0.5
no (n=387)	31.5		35.4		40.1		75.7		68.5		45.7	
Living alone												
yes (n=175)	30.3	0.6	26.3	0.3	32.0	0.7	80.0	0.3	66.9	0.5	47.4	0.9
no (n=939)	28.4		30.0		33.3		76.3		64.1		47.2	
Self-declared household financial status												
good (n=524)	29.8	0.8	31.7	0.3	35.7	0.08	81.1	**0.002**	66.6	0.4	52.7	**0.001**
moderate (n=436)	28.0		27.1		32.6		74.8		62.8		44.0	
bad (n=154)	27.3		28.6		26.0				62.3		37.7	
History of mental disorders in the family												
yes (n=235)	36.2	**0.01**	38.7	**<0.001**	46.0	**<0.001**	81.7	**0.04**	71.1	**0.02**	57.4	**0.001**
no (n=879)	26.7		27.0		29.7		75.5		62.8		44.5	

Bold values are statistically significant.

### 3.3 Factors associated with the level of knowledge of mental disorders

A clear urban–rural gradient was observed, with respondents living in rural areas being least likely to report good knowledge of mental disorders, potentially due to reduced access to mental health information and services. In multivariable logistic regression (**Table 7**), age <60 years (p<0.05), living in cities over 20,000 residents (p<0.05), having higher education (aOR=1.77 [1.23-2.55]; p=0.002) and history of mental disorders in the family (aOR=2.69 [1.86-3.89]; p<0.001) were significantly associated with rather good/very good level of knowledge on mental disorders. Lower awareness among individuals with moderate household economic status may reflect limited health literacy, fewer contacts with mental health professionals, and lower exposure to public health campaigns, particularly outside urban centers. Respondents with moderate economic status were less likely to declare very good/rather good level of knowledge on mental disorders (aOR=0.53 [0.30-0.93]; p=0.03). There was no significant (p>0.05) impact of gender, professional status, relationship status, having children, and living conditions on self-reported level of knowledge on mental disorders. (**Table 7**).

**Table 7:** Factors associated with self-reported level of knowledge on mental disorders.

Variable	Self-reported good or very good level of knowledge on mental disorders					
	%	p	Bivariable Logistic Regression		Multivariable Logistic Regression	
			OR (95%CI)	p	aOR (95%CI)	p
Gender						
female (n=609)	18.6	**0.04**	1.42 (1.02-1.96)	**0.04**	1.25 (0.88-1.79)	0.2
male (n=505)	13.9		Reference		Reference	
Age [years]						
18-29 (n=151)	30.5	**<0.001**	6.85 (3.84-12.22)	**<0.001**	6.35 (3.15-12.81)	**<0.001**
30-39 (n=217)	22.1		4.44 (2.53-7.80)	**<0.001**	3.65 (1.89-7.08)	**<0.001**
40-49 (n=213)	18.3		3.50 (1.96-6.25)	**<0.001**	2.92 (1.53-5.56)	**0.001**
50-59 (n=217)	14.3		2.61 (1.43-4.75)	**0.002**	2.24 (1.16-4.33)	**0.02**
60+ (n=316)	6.0		Reference		Reference	
Place of residence						
rural area (n=423)	10.4	**<0.001**	Reference		Reference	
city <20,000 residents (n=143)	16.1		1.65 (0.96-2.85)	0.07	1.60 (0.90-2.86)	0.1
city 20,000-99,999 residents (n=218)	20.6		2.24 (1.43-3.52)	**<0.001**	2.21 (1.37-3.57)	**0.001**
city 100,000-499,999 residents (n=187)	19.8		2.13 (1.32-3.42)	**0.002**	2.02 (1.21-3.36)	**0.01**
city ≥ 500,000 residents (n=143)	23.8		2.69 (1.64-4.41)	**<0.001**	2.23 (1.31-3.81)	**0.003**
Educational level						
higher (n=508)	21.5	**<0.001**	1.96 (1.42-2.71)	**<0.001**	1.77 (1.23-2.55)	**0.002**
less than higher (n=606)	12.2		Reference		Reference	
Professional status						
active (n=685)	19.6	**<0.001**	1.89 (1.33-2.68)	**<0.001**	1.19 (0.78-1.82)	0.4
passive (n=429)	14.4		Reference		Reference	
Relationship status						
married or informal relationship (n=779)	15.1	0.08	0.74 (0.53-1.04)	0.08		
single (n=335)	19.4		Reference			
Having children						
yes (n=727)	13.8	**<0.001**	Reference	**0.001**	Reference	
no (n=387)	21.4		1.71 (1.24-2.36)		0.95 (0.63-1.42)	0.8
Living alone						
yes (n=175)	20.0	0.2	1.34 (0.89-2.01)	0.2		
no (n=939)	15.8		Reference			
Self-declared household financial status						
good (n=524)	21.2	**<0.001**	1.32 (0.83-2.12)	0.2	1.02 (0.61-1.71)	0.9
moderate (n=436)	10.6		0.58 (0.35-0.98)	**0.04**	0.53 (0.30-0.93)	**0.03**
bad (n=154)	16.9		Reference		Reference	
History of mental disorders in the family						
yes (n=235)	29.8	**<0.001**	2.88 (2.04-4.05)	**<0.001**	2.69 (1.86-3.89)	**<0.001**
no (n=879)	12.9		Reference		Reference	

OR – odds ratio; aOR – adjusted odds ratio Bold values are statistically significant.

## 4 Discussion

This is the first nationwide cross-sectional survey on public awareness of mental disorders and their risk factors among adults in Poland. Findings from this study revealed significant gaps in public knowledge on mental disorders. Depression was the most recognized mental disorder. Traumatic experiences like physical, psychological, sexual violence, war, or natural disasters were the most recognized risk factors for mental disorders, with low recognition of lifestyle-related mental disorders risk factors. Out of 10 different sociodemographic variables included in multivariable regression analysis, age, place of residence, educational level and family history of mental disorders were the only factors associated with higher levels of knowledge on mental disorders.

Building public awareness of mental disorders is an important element of national mental health strategies and translates into the possibility of early referral to psychiatric facilities for people suspected of having a mental disorder (22). People who are aware of mental disorders may be more likely to seek professional help at the early stage of a mental crisis. Moreover, people with a high level of awareness of mental disorders may be more likely to their relatives, friends, or co-workers who are affected by mental disorders or mental crises.

In this study, only 16.4% of respondents declared a very good or rather good level of knowledge on mental disorders. This observation may result from the fact that the National Mental Health Program in Poland, is mostly focused on the organization of mental health services, with a limited number of nationwide educational programs (14). Depression was the most recognized mental disorder which may result from the fact, that significant part of the educational campaigns on mental disorders, carried out by public organizations or NGOs in Poland was focused on depression (23). Moreover, talks on depression and its symptoms are often presented in mass media.

Awareness of risk factors of mental disorders is necessary to modify lifestyle behaviors and decrease the risk of mental disorders. Moreover, people who are at higher risk of mental disorders due to non-modifiable risk factors (like genetic predisposition or experiences in the past) may be more likely to seek help at an early stage of mental health deterioration, as they are more aware of their potential mental disorder risks. Lack of awareness of mental disorders among adults in Poland may also lead to misunderstanding and false social perception of people with mental disorders. For example, people with chronic diseases like diabetes are at higher risk of depression, but due to low awareness of the risk factors for mental disorders, relatives and friends of patients with chronic diseases may not be aware that chronic diseases may evoke changes in behaviors and mental disorders like diabetes (24). Environmental pollution was the least recognized risk factor for mental disorders. Numerous studies have shown that environmental pollutants have an impact on human mental health through different biological pathways (25). In numerous cities in Poland, air pollution levels exceed WHO air quality standards (26). Lack of awareness of the link between environmental factors and mental health requires implementation of urgent educational activities.

Urban areas contribute to higher awareness of mental disorders through multiple mechanisms that differ significantly from those available in rural settings. One major factor is the greater accessibility of mental health services in cities, owing to the higher concentration of healthcare facilities and trained professionals (27). Additionally, urban environments often host more educational programs and public awareness campaigns, frequently supported by NGOs and public health initiatives (28). Urban design also plays a role; interventions aimed at creating restorative environments can enhance mental health literacy and offer psychosocial support (29). Moreover, tele-psychiatry is more accessible in urban areas, facilitating timely diagnosis and treatment, in contrast to its limited availability in rural regions (30). Finally, urban populations typically exhibit higher levels of education and income, both of which are positively correlated with improved knowledge of and attitudes toward mental health issues (31).

Out of 10 different sociodemographic factors, only 4 were significantly associated with a higher level of knowledge of mental disorders. Individuals <60 years of age were more likely to declare a higher level of knowledge of mental disorders. This observation may result from the fact that younger generations are more likely to focus on mental health and well-being. This observation may result from the fact that younger generations are more likely to focus on mental health and well-being (32). Moreover, younger adults may be more likely to hear about mental health on social media or in the workplace (33, 34). Living in cities with > 20 thousand residents was also associated with a higher level of knowledge of mental disorders. This observation suggests that current educational campaigns on mental disorders do not sufficiently reach the inhabitants of villages and small towns. This observation may also impact the social perception of people with mental disorders who live in small local communities (35). Moreover, those who live in rural areas or small towns may be less likely to seek professional health during a mental health crisis. Local governments should urgently address this gap through educational and information campaigns targeted at local communities. Educational levels often affect health disparities (36). In general, those with higher education often present higher health literacy skills (37). Findings from this study also confirmed that higher education was associated with a higher level of knowledge of mental disorders. In this study also the family history of mental disorders was associated with a higher level of knowledge of mental disorders. The presence of disease in the family and personal history related to relatives with mental disorders may impact individual attitudes toward mental disorders.

There is a lack of other studies on public awareness of mental disorders in Poland, so direct comparisons are difficult. Uddin et al. showed that awareness of mental health conditions in a rural district in Bangladesh appeared to be very limited and the lowest knowledge on mental disorders was among females, older adults, people with low socioeconomic status and primary levels of education (38). More nationwide studies on public awareness of mental disorders are needed.

The study has several practical implications. First, nationwide data on public awareness of mental disorders and their risk factors may be used by healthcare professionals and policy makers to implement educational campaigns and may be included in the national mental health strategy. Second, sociodemographic differences in public awareness of mental disorders and their risk factors point out groups with the lowest level of knowledge on mental disorders, that should be targeted during the educational activities. Third, this study showed that there is an urgent need to educate adults in Poland on lifestyle-related risk factors for mental disorders, that may result in increase of adults who implement mental disorders prevention measures.

This cross-sectional survey has several limitations. Data were self-declared so recall bias may occur. The level of knowledge on mental disorders was based on one simple question with 5-point Likert scale and was not verified in more advanced tests or scales. This study was limited to 9 different mental disorders and 12 most common risk factors, so only selected mental disorders were assessed in this study. Data on mental disorders in the family were self-declared and was not crossed with medical documentation. CAWI method was used so only respondents with Internet access could participate in this study. The psychometric properties of the self-reported questionnaire used in this study, including its reliability and validity, have not been formally assessed, however, the pilot restudy was conducted on eight adults who completed the questionnaire twice, with a one-week interval. Based on the results of this pilot, one question and three response options were modified to improve the consistency and clarity of the questionnaire.

## 5 Conclusions

The level of knowledge on mental disorders among adults in Poland is low. Significant gaps in awareness of most common mental disorders and their risk factors (especially lifestyle-related risk factors) were found. Educational campaigns on mental disorders should be particularly targeted at older adults, citizens of rural areas of small cities, and those without higher education.

## Data Availability

The raw data supporting the conclusions of this article will be made available by the authors, without undue reservation.
